# Yoga Pose Estimation and Feedback Generation Using Deep Learning

**DOI:** 10.1155/2022/4311350

**Published:** 2022-03-24

**Authors:** Vivek Anand Thoutam, Anugrah Srivastava, Tapas Badal, Vipul Kumar Mishra, G. R. Sinha, Aditi Sakalle, Harshit Bhardwaj, Manish Raj

**Affiliations:** ^1^Computer Science Engineering Department, Bennett University, Greater Noida, India; ^2^MIIT, Mandalay, Myanmar; ^3^School of Computer Science and Engineering, Galgotias University, Greater Noida, India; ^4^Computer Science Engineering Department, Bennett University, Greater Noida, India

## Abstract

Yoga is a 5000-year-old practice developed in ancient India by the Indus-Sarasvati civilization. The word yoga means deep association and union of mind with the body. It is used to keep both mind and body in equilibration in all flip-flops of life by means of asana, meditation, and several other techniques. Nowadays, yoga has gained worldwide attention due to increased stress levels in the modern lifestyle, and there are numerous methods or resources for learning yoga. Yoga can be practiced in yoga centers, through personal tutors, and can also be learned on one's own with the help of the Internet, books, recorded clips, etc. In fast-paced lifestyles, many people prefer self-learning because the abovementioned resources might not be available all the time. But in self-learning, one may not find an incorrect pose. Incorrect posture can be harmful to one's health, resulting in acute pain and long-term chronic concerns. In this paper, deep learning-based techniques are developed to detect incorrect yoga posture. With this method, the users can select the desired pose for practice and can upload recorded videos of their yoga practice pose. The user pose is sent to train models that output the abnormal angles detected between the actual pose and the user pose. With these outputs, the system advises the user to improve the pose by specifying where the yoga pose is going wrong. The proposed method was compared to several state-of-the-art methods, and it achieved outstanding accuracy of 0.9958 while requiring less computational complexity.

## 1. Introduction

Like every exercise, it is most important to practice yoga poses accurately as any abnormal posture is not productive and tends to cause harm. This suggests having an instructor around while performing yoga. It is not always possible to have an instructor or to join yoga classes with nowadays lifestyle. An AI-based system helps to identify yoga poses and gives feedback or suggestions to users. These instructions help users improve their poses so that it is productive and not detrimental. The challenges in this project are key points should be detected without any missing points and models should work properly even when body parts are overlapped. Suggestions should be given accurately since slight changes may cause harmful results. The poses in datasets used for this project should be done by experts. Models should accurately classify poses, even though they are nearly the same poses with a slight difference in them.

Automated self-training methods for sporting activities can help players enhance their performance and reduce the risk of injuries. Many researchers have developed computerized systems for evaluating exercise-related activities such as football player rankings, handball strikes, volleyball, sprints, jumping, and other athletic activities. Patil et al. [1] proposed a ‘Yoga Tutor' project that uses accelerated robust characteristics to make the distinction in postures between a learner and a professional (SURF). Wu et al. [2] presented a picture and text-based intelligent systems for yoga, but they did not look at the posture of the practitioner. Chen et al. [3] used a features-based method to create a self-training system that recognized yoga exercises. It makes use of a Kinect to capture the person's body contour and create a body map. To obtain a descriptor for the human position, a star skeleton was employed for quick skeletonization. In reference [4], a yoga identification system that is based on a Kinect and the AdaBoost classification with a 94.78 accuracy score is proposed for six asanas. They are, however, utilizing a depth sensor-based camera that is normally not obtainable. Using convolutional neural network (CNN) and stacked autoencoder (SAE) methods, Mohanty et al. [5] implemented an image recognition approach for detecting Indian traditional dance and yoga postures from photographs. They did, though, only analyze their competence on still photos, never on videos. Since the introduction of DeepPose by Toshev et al. [6], the traditional skeletonization methodology has been supplanted by deep learning-based technologies. DeepPose is leading the charge away from traditional techniques and toward deep network-based approaches. It directly regresses on joint coordinates using deep neural network-based regressors. It anticipates a person's activities and also forecasts the location of hidden body parts. However, their approach has difficulty with localization.

In recent years, there are related works on yoga pose detection and classification. [7] Keypoint detection methods used are OpenPose [8], PoseNet [9], and PifPaf [10]. To detect human pose, many factors will be considered such as surroundings, human interactions, and variations in clothing [11]. Deep learning methods they used for pose classification are multilayer perceptron, recurrent neural network, long short-term memory (LSTM) [12, 13], and convolutional neural network. Limitations in the above works are that features (key points) are not scaled and are unable to find a pattern for human poses of different distances from the camera. Previous methods that used joint angles as features are rotational invariant (even if joints are rotated, the angles between them are not changed) [14, 15].

**Figure 1:**
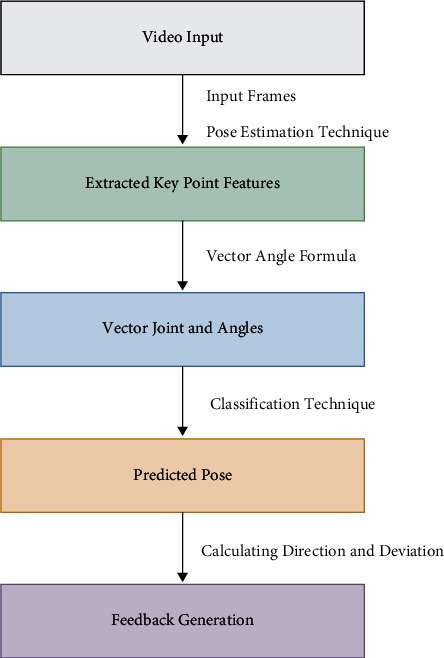
The figure illustrates the overview of the proposed method.

In the proposed work for abnormal pose detection, the research used networks that classify yoga poses and calculate deviation from the already calculated expert pose. This project mainly focuses on preprocessing datasets to extract new features like angles between body parts and how they improve accuracy compared to traditional pose features and by filling missing values. This system uses classification networks like multilayer perceptron and hyperparameters tuning to achieve good accuracy. The first phase of the project talks about related works in this field, continued with the description of the dataset the study used and preprocessing techniques. How angles are extracted is discussed in preprocessing. Then, the project focused on a pose estimation code and MLP (multilayer perceptron) training for the yoga classification. At last, model evaluation metrics and how suggestions are constructed for users are discussed. The overview of the proposed method is shown in Figure 1.

The remaining paper is organized as follows. The related work for yoga pose estimation is discussed. The datasets are outlined, and the section expounds on the proposed methodology to extract key points and features, classification, and feedback generation. The implementation details, evaluation measures of the proposed approach, and the runtime analysis are discussed. Finally, the conclusion and future scope are outlined.

## 2. Related Work

Human activity recognition has been employed in a variety of applications, including robotics and computer engineering. References [16, 17] use randomized trees (random forests) for detecting human activities with the help of sensors. Reference [18] uses hidden Markov models and recognized body parts for human activity recognition. This method is used for the recognition of 6 home activities, which achieved an accuracy of 97.16 percent. This method is used at smart homes for monitoring services. [19] It uses environmental background sounds for human activity recognition where wearable sensors that detect sounds are used, which achieved an accuracy of 96.9 percent.

Significant work has been done in developing automated systems, which analyze yoga and sports activities like basketball [20] and cycling [21]. [1] An automated system for naive users to perform yoga and compare with expert yoga videos uses a Speeded Up Robust Features (SURF) algorithm using only contour information, which may not be sufficient. [4] An automated project for yoga pose detection using kinetic sensors and an AdaBoost classifier achieved 94.8% accuracy. Another system presented in reference [3] for 3 yoga poses achieved 82.84% accuracy. [5] The system used deep learning techniques for the classification of yoga poses. In traditional machine learning, [22] models require extracted features and engineering, but deep learning understands data and extracts features. [2] A self-instructed system is built for the yoga pose using star skeleton computation. To extract the body contour from the user body map, Kinect is used and achieved an accuracy of 99.33 percent. [23] It used hash-based learning to extract human pose from the pressure sensor. These sensors may not be feasible always to carry, so in the proposed system, no sensors are used.

The pose estimation used is in OpenPose and used the hybrid model CNN with LSTM to classify yoga poses, and this model incorporates feature extraction. Also, it compared basic CNN models with a hybrid model, and machine learning models were compared with deep learning models. Evaluation metrics used are classification score and confusion matrix. SM achieved a test accuracy of 0.9319, CNN achieved 0.9858, and the hybrid model CNN with LSTM achieved 0.9938. There are many keypoint detection methods like OpenPose, PoseNet, and PifPaf. OpenPose [8] invented in CMU and CNN-based architecture is used to obtain key points. OpenPose uses VGG-19 for the extraction of features from images. 18 confidence maps were detected by the first branch (initial layers). The second branch is used for predicting the association between body parts.

PoseNet [9] is similar to OpenPose, which can extract human pose. All these key points are indexed with confidence levels, 1 being greatest and 0 being lowest. PoseNet does not depend on the size of images; even though images are downscaled, the pose is extracted. [24] The encoder generates an encoding vector, localizer converts encoding to a localization feature vector, and regressor is utilized to regress the final pose. PifPaf [10] is based on a bottom-up approach for extracting human pose. A Part Intensity Field is used for body parts localization, and a Part Association Field is used for body parts association; these two combine for the entire body pose. The architecture used is ResNet.

The features used by these models are 18 key points; the input size is 36 *x* and *y* coordinates of each key point. Models will train with more accuracy if features are extracted from these key points. In the project, 12 features say angles are extracted of 12 different joints, which are used as an input to models. Previous methods [25–27], which used joint angles as features, show that human activities and angle motion sequences are related. These angle features, as they are scalable, have more information than key points. In reference [25], it is shown that angles between elbows, shoulders, knees, and crotch contribute more information for 3D human activity detection. In reference [28], angle pairs for hip bones are added, and in reference [27], for standing and walking actions, features like right knee, left knee, and elbow provide more information.

In references [28, 29], features used are angles between joints so that features are scaled. In reference [29], features mainly used are hip and knee angles. Angles at the hip joints are angles made by the shoulders and knees, and angles at the knees are angles made by the hips and ankles. These features give more insight than key points because at any distance from the camera, the angles extracted will be the same, but not key points as they are not scaled. In reference [28], angles are calculated concerning a reference key point in a 3D space. But in both these cases, these features are rotation invariant. In the proposed system, angles with respect to the *x*-axis, that is, ground, are considered. There are 12 joints where every joint connects 2 key points, so 12 features (angles) are present entirely. Suppose, a and b are two key points, then the angle made by joint ab with the *x*-axis acts as a feature.

## 3. Methodology

In this paper, a deep learning-based yoga pose estimation methodology presented in algorithm 1 is proposed to detect correct yoga poses and provide feedback to improve the yoga posture. The proposed approach has been done on Nvidia DGX V-100 and consists of three main steps:

1. **Feature extraction**: videos or images are given as input to the model, and frames are extracted at regular intervals from videos and sent to Keras multiperson pose estimation to extract key points. From these key points, 12 joint vectors are calculated. For all these 12 joints, angles between the *x*-axis and joints are found, respectively.
2. **Classification**: these angles are sent to the classification model to classify the pose among 6 yoga poses. These angles are compared with an array of 12 angles of the classified pose. This array contains average angles of 12 joints from the dataset.
3. **Feedback generation**: the differences are calculated, respectively, for every angle, and suggestions are revealed for every angle. Based on the sign of difference, whether to rotate joints in clockwise or anticlockwise direction is given as feedback output.

**Figure 2:**
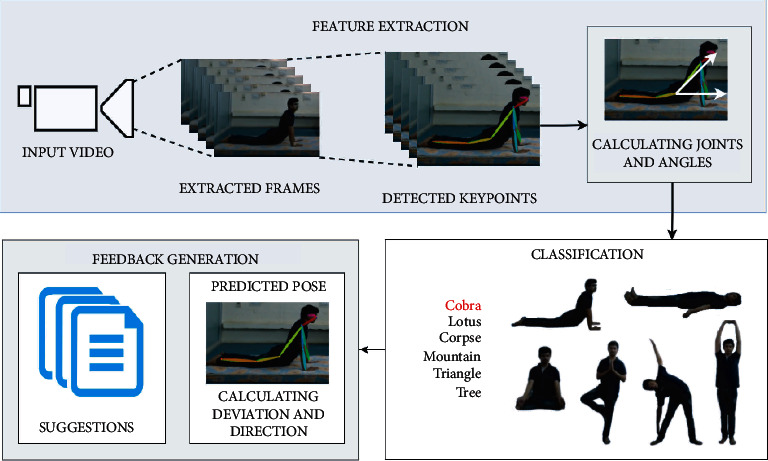
A schematic diagram of the proposed approach for correct yoga pose estimation and feedback generations for incorrect posture.

The proposed approach is represented schematically in Figure 2, and further explanations of each step are provided in the following sections.

### 3.1. Datasets

**Figure 3:**
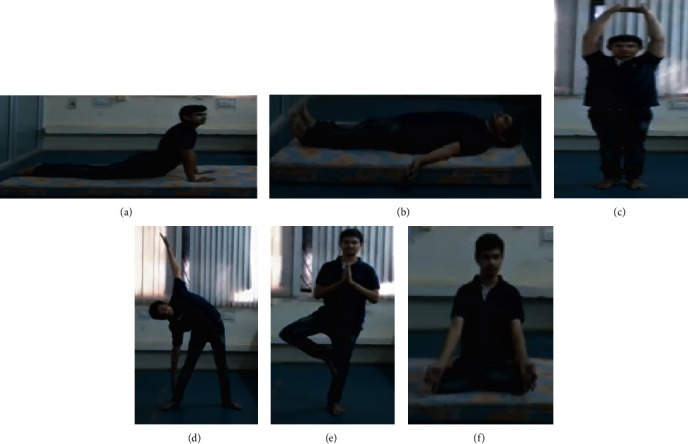
Sample frames of every yoga pose: (a) Cobra, (b) Corpse, (c) Mountain, (d) Triangle, (e) Tree, and (f) Lotus pose.

The proposed methodology is examined on a publicly available, online, open-source collection [30] dataset. This dataset includes 6 yoga poses, namely, Cobra (Bhuj), Tree (Vriksh), Mountain (Tada), Lotus (Padam), Triangle (Trik), and Corpse (Shav). Total videos of the 6 poses are 70, and total instances combining the 6 poses are 350. These videos are recorded in a room using the camera from a distance of 4 meters; the frame per second rate is 30. To have robust trained models, individuals performed these poses with few variations.  Table 1 summarizes the statistics of the dataset in terms of video count, duration of each activity class in seconds, and the number of persons for each yoga poses separately, and some sample frame of every pose is depicted in Figure 3.

This dataset is used for training (320 instances) and validation (30 instances). A separate dataset is created from these videos at different time intervals for testing. This separate dataset contains a total of 30 instances, 5 for each pose.

### 3.2. Real-time Multiperson Pose Estimation

Human pose estimation is one of the important challenges of computer vision and has made many advancements in the last few years. 3D pose estimation evolved from a 2D pose and single-person pose estimation to multiple-people pose estimation. Pose estimation algorithms generally detect body points, link body points, and output their key points. These key points have *x* and *y* coordinates of every body point, which helps in many computer vision problems like surveillance-assisted living, gym-pose analysis or any sports analysis, and activity recognition.

**Figure 4:**
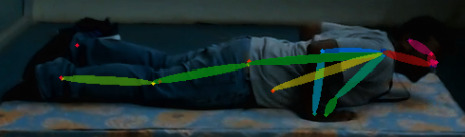
Person pose estimation for the Bhuj pose with different confidence levels.

This pose estimation extracts 18 body key points where every point consists of *x* and *y* coordinates of body points. This code outputs one dictionary and one 2D array. The dictionary contains keys as body parts and values as their coordinates; in an array, if many values are detected for a key in the dictionary, then all this information is present with their corresponding confidence levels. In the dictionary, only the first detected body point is present even if the confidence level is low compared with other values. So, the code needs to be changed so that values are chosen based on a high confidence level. For example, in the Bhuj pose, for the right wrist, two body points are detected with different confidence levels (Figure 4).

### 3.3. Feature Extraction

**Figure 5:**
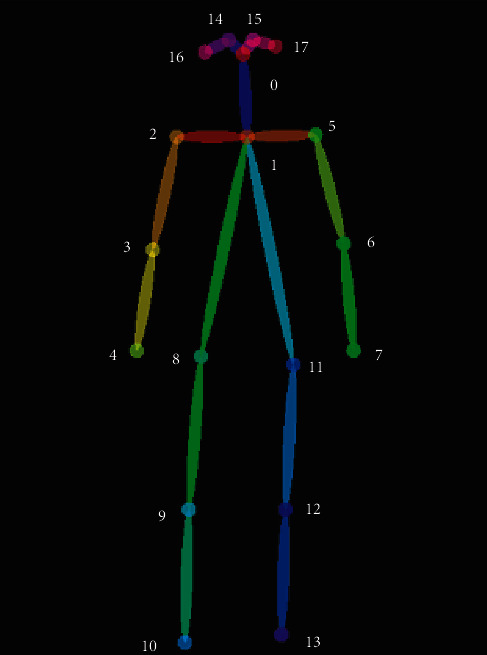
Extracted key points from a frame by the pose estimation method [7].

To extract key points for pose estimation, Keras real-time multiperson pose estimation is utilized [7, 8]. This pose estimation is run on every video, frames are extracted for every 2 seconds, and pose is calculated for 5 consecutive frames of each video, which results in 350 instances for 70 videos. Every pose outputs an array of 18 key points where every point consists of *x* and *y* coordinates.  Figure 5 shows key points extracted from a frame by the pose estimation code.

**Figure 6:**
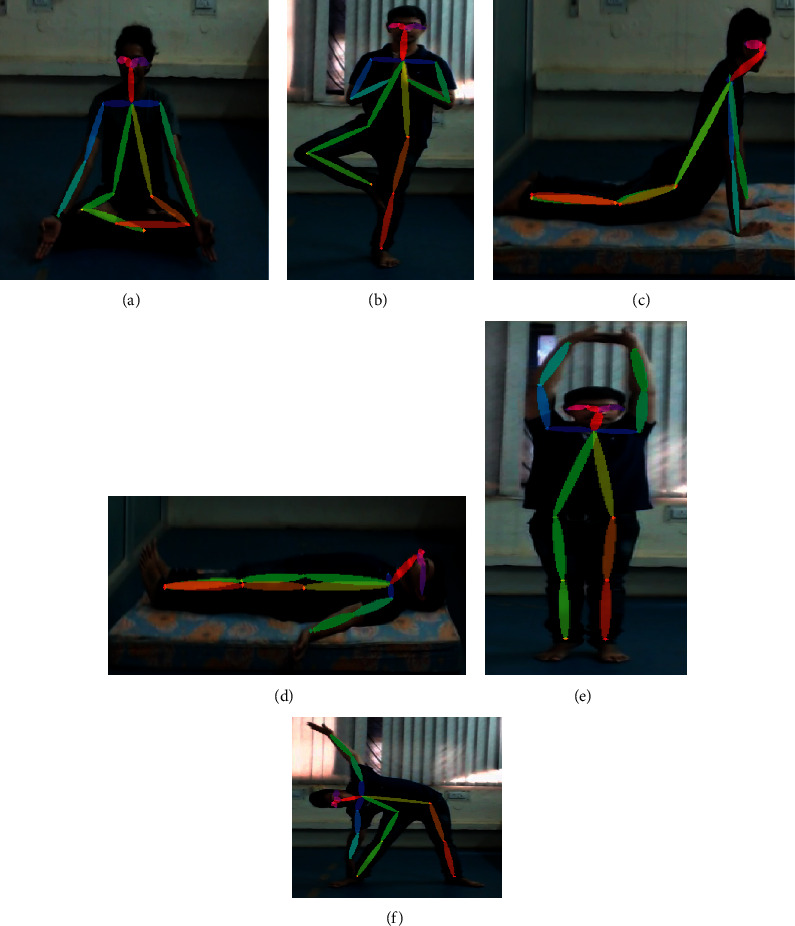
Demonstration of key points extraction on all 6 yoga poses: (a) Lotus pose, (b) Tree pose, (c) Cobra pose, (d) Corpse pose, (f) Triangle pose, and (e) Mountain pose.

The research work has used 320 instances for training. While detecting poses for a person, many key points are being detected with different confidence levels. Keras pose estimation works in such a way where it includes the first key point detected without taking into consideration confidence intervals. In this paper, a few modifications were done to the Keras pose estimation to consider key points of highest confidence levels. With these *x* and *y* coordinates, the study extracted features like angles between body joints and with the ground so that models will be trained to achieve good accuracy. Utmost priority is given to these instances so that there will be no abnormality data given as input.  Figure 6 depicts pose estimation on all 6 yoga poses.

**Figure 7:**
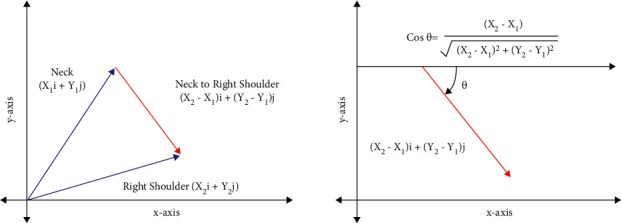
The coordinate vector and angle calculation representation of neck and right shoulder vectors.

Every extracted point is treated as a vector-connecting origin. In body points, nose, ears, and eyes features are not considered as they are not important features, and the features whose confidence score is less than 0.3 are also not considered in order to consider the joints that are accurately visible. So, the number of vectors present is 13. In total, the feature set has 12 joints without nose, ears, and eyes. The 12 joints are neck to the right shoulder, right shoulder to the right elbow, right elbow to the right wrist, neck to the left shoulder, left shoulder to the left elbow, left elbow to the left wrist, neck to the right hip, right hip to the right knee, right knee to the right ankle, neck to the left hip, left hip to the left knee, and left knee to the left ankle. From these 13 vectors, 12 joints can be obtained by subtracting vectors. Suppose, body point neck and right shoulder are (*x*_1_, *y*_1_) and (*x*_2_, *y*_2_), respectively. Then, their vectors are *x*_1_*i*+*y*_1_*j* for the neck and *x*_2_*i*+*y*_2_*j* for the right shoulder. To get a vector for the joint neck and right shoulder, subtract the neck vector from the shoulder vector, which is (*x*_2_ − *x*_1_)*i*+(*y*_2_ − *y*_1_)*j* as shown in Figure 7. But, −1 should be multiplied with (*y*_2_ − *y*_1_) because origin in images is present at the top left corner, which is different from the bottom left corner. So, the vector for the joint is (*x*_2_ − *x*_1_)*i*+(−1)*∗*(*y*_2_ − *y*_1_)*j*. In this way, 12 vectors for 12 joints are obtained and the angles they are making with the *x*-axis need to be calculated. Suppose, the angle made by a vector with the *x*-axis is theta, then cos(*θ*) for the vector (*x*_2_ − *x*_1_)*i*+(*y*_1_ − *y*_2_)*j* is (*x*_2_ − *x*_1_)/(*x*_2_ − *x*_1_)+(*y*_1_ − *y*_2_). With this method, 12 angles for 12 different vectors for 12 joints are obtained. So, the feature set has 12 columns.

These angles extracted are scaled and rotation variants. Different poses varying in distances from the camera need to scale key points to train models to achieve high accuracy. But, when angles are used as features, varying distances does not have any need to scale any feature. For example, if the joint left ankle and left knee make 90 with the ground, considering for different distances from the camera, the angle made by this joint will be the same at any distance. If points are rotated, suppose the joint left ankle and left knee rotate by any slight angle, then the angle made by the *x*-axis will be varied. In reference [29], angles made at the hip will not change if all 3 key points (shoulders, hips, and knees) rotate at the same angle. In reference [28], if two key points are swapped, then the angle made concerning the reference point will not change. Hence, angles used in references [28, 29] are rotation invariant, and in the system, angles are rotation variants.

### 3.4. Feedback Generation

In the dataset, average values or angles are calculated for every pose by considering all poses done by everyone. When images are given as input to the model, the trained model classifies the pose with which it aligns. The angles extracted from the image are compared with the average values calculated. The differences between these angles are calculated, respectively, that is, 12 values are calculated. To give suggestions, two parameters are needed—how much the pose is deviated from the original and in what direction. The magnitude of these 12 difference values tells by how much one must correct his pose, and for the direction, the researchers used the sign whether it is positive or negative, which tells us to rotate the joints in either clockwise or anticlockwise direction. With this method, suggestions are given to users for every joint.

## 4. Results

Neural networks (MLP) are built using 3 types of layers, namely, input layer, hidden layers, and output layer. There can be any number of hidden layers based on the complexity of training data. If hidden layers are few, the model may underfit training data, and if they are more, the model may overfit. MLP is a fully connected neural network, that is, every node is connected to every other node in consecutive layers in the neural network. Generally, these networks are utilized for supervised training where for every input data, there is a corresponding output label or class.

**Figure 8:**
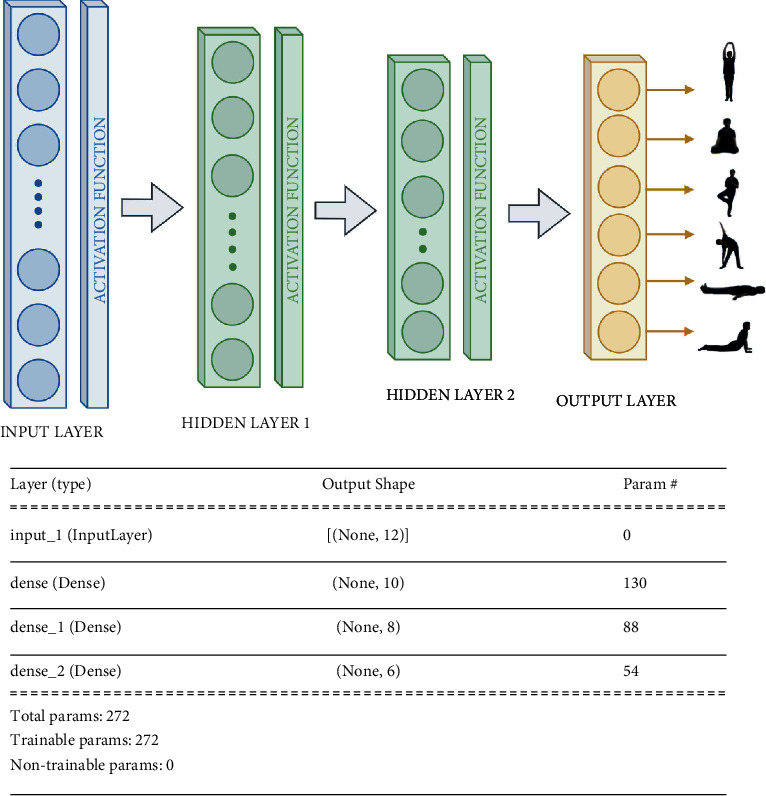
Neural network model architecture.

**Figure 9:**
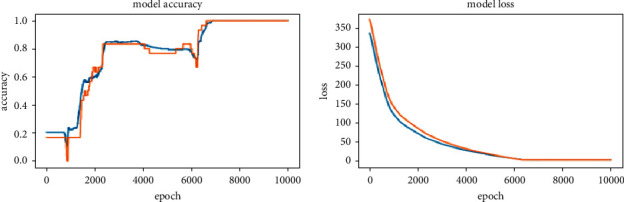
Graphs of accuracy and loss for training and validation datasets.

Multilayer Perceptron (MLP) is used for human pose classification [31]. In this paper, angles between key points have been computed and passed as input for MLP. In the project, the input data length is 12 and has 6 classes to classify these labels, so the output layer length is 6.  Figure 8 describes the input layer size as 12, 1st hidden layer size is 10, 2nd hidden layer size is 8, and output layer size is 6. In total, there are 350 instances, for the training of which 320 instances are used, whereas for every pose, 5 instances are used for validation. The batch size used for training is 20, and the number of epochs is 10000.  Figure 9 shows the graphs of accuracy and loss for training and validation datasets.

Both training and validation datasets have many ups and downs in accuracy till the 6900 epoch and attained an accuracy of 0.9958 at the 6900 epoch. From 6900 to 10000 epochs, loss of training and validation decreased gradually, which results in the training model classifying with high confidence. From epoch 0 to 10000, the loss of validation and training datasets decreased gradually. From the training and validation accuracy obtained, it can be inferred that the model is not overfitting. The loss function used is categorical cross-entropy since the research is classifying input features into one of the 6 labels. An AdaDelta optimizer is used based on the adaptive learning rate to address two drawbacks: (1) decay of learning rates and (2) selection of the global learning rate. The activation function used for the last layer is softmax since it outputs confidence levels for all labels. The one with the highest confidence is the predicted label.

Table 2 represents the accuracy result of the experimented models, SVM obtained accuracy results of 0.9319, CNN obtained accuracy results of 0.9858, and CNN + LSTM achieved accuracy results of 0.9938. MLP power in the system is substantially smaller than CNN and CNN + LSTM, but it obtained an accuracy of 0.9958 with modified features.

To examine the effectiveness of the proposed methodology, a confusion matrix is utilized that describes the classification model performance for all instances in terms of accuracy as per equation (1). Classification accuracy is also known as classification score, which is the ratio of correct classifications and total instances.

**Figure 10:**
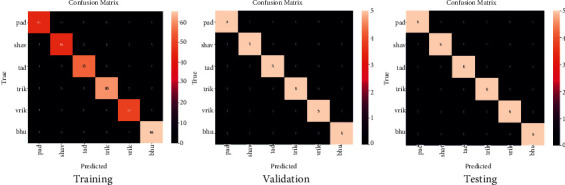
Confusion matrices of training, validation, and testing datasets. (a) Training, (b) validation, and (c) testing.

**Figure 11:**
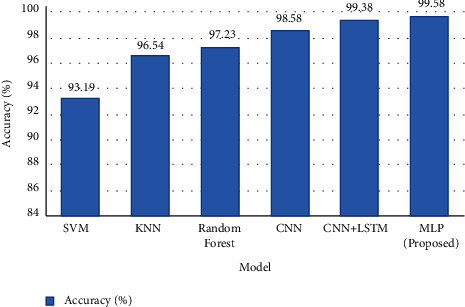
The graph illustrates the plot of different competitive models.

The confusion matrix used in the study has 6 labels, so the result evaluation has a 6 × 6 confusion matrix. The *i*_th_ row represents the actual class, while the *j*_*th*_ column represents the predicted class of the proposed data.  Figure 10 depicts the confusion matrices of training, validation, and testing datasets. In the confusion matrix of the training, validation, and training dataset, the total number of instances are 320, 30, and 30, respectively. It can observe that all the samples are predicted correctly, which results in an accuracy of 0.9958 for all cases.  Figure 11 represents the plot for different competitive models.

(1) $$\begin{array}{c} \text{Classification Accuracy}=\frac{\text{Total Number of Correct Classifications}}{\text{Total Input Samples}}. \end{array}$$

## 5. Runtime Analysis

The approaches presented in this research are based on deep learning to detect incorrect yoga posture and also advise the user to improve the posture. In this research, extraction of key points using a pose estimation technique, computation of vectors for each joint, and the angle between the vectors for adjacent joints are categorized as features. Following that, these features were fed into the classification techniques, and later, the feedback for the correctness of the yoga pose is generated. Therefore, the runtime is divided into three parts: (1) extraction and computation of features time for every frame, (2) classification, and (3) feedback generation time of categorizing yoga pose per frame. The runtime for the extraction and computation of features remains constant for each method. The runtime analysis is carried out on the Xeon(R) CPU E3-1240 v5 and NVIDIA GeForce GTX-1080.

Table 3 presents the mean average runtime per frame together with the standard deviation of the experimental methodology. The time is presented in milliseconds. It incorporates the time taken per frame on feature extraction and classification with feedback development.

## 6. Conclusions

The approaches presented in this research are based on deep learning to detect incorrect yoga posture and also advise the user to improve the pose by specifying where the yoga pose is going wrong. In the proposed system, the users can select the desired pose for practice and can upload recorded videos of their yoga practice pose. The research has extracted monitoring activities angles and used them as a feature as they are scaled. In some cases, if key points are rotated then angles are not changing, which does not deliver good results. In this system, angles with the ground are considered but not between joints, so if there is any slight rotation of key points, then angles are changed. With these features, multilayer perceptron is trained to achieve an accuracy of 0.9958 for testing datasets. In existing research, SVM achieved a test accuracy of 0.9319, CNN achieved 0.9858, and CNN + LSTM achieved 0.9938. In the system, MLP power is much lower than CNN and CNN + LSTM but achieved an accuracy of 0.9958 with modified features. When compared to existing techniques, the experimental results show promising results. The proposed approach maintains low computational complexity, can be applied to someone's busy life for self-yoga learning, and can detect incorrect yoga posture to avoid chronic problems.

### 6.1. Future Scope

The proposed system is confined to 6 yoga poses, where there are a total of more than 80 yoga poses. The proposed dataset can be expanded by adding required yoga pose key points. The technology may also be used to make real-time predictions and self-training on a mobile device. There are several instances of real-life applications in which a single individual posture evaluation will not be enough; for example, a pose estimate in crowded environments will need to detect and recognize the pose of each participant. To include many poses and to get model works on many poses (classifying many poses) is challenging enough. Keras pose estimation influences the performance of the model; steps should be taken to get key points when body parts are overlapped or missing to achieve better results. This method to extract angles as features can be used for other applications like activity detection and sports activity monitoring.

## Figures and Tables

**Algorithm 1:**
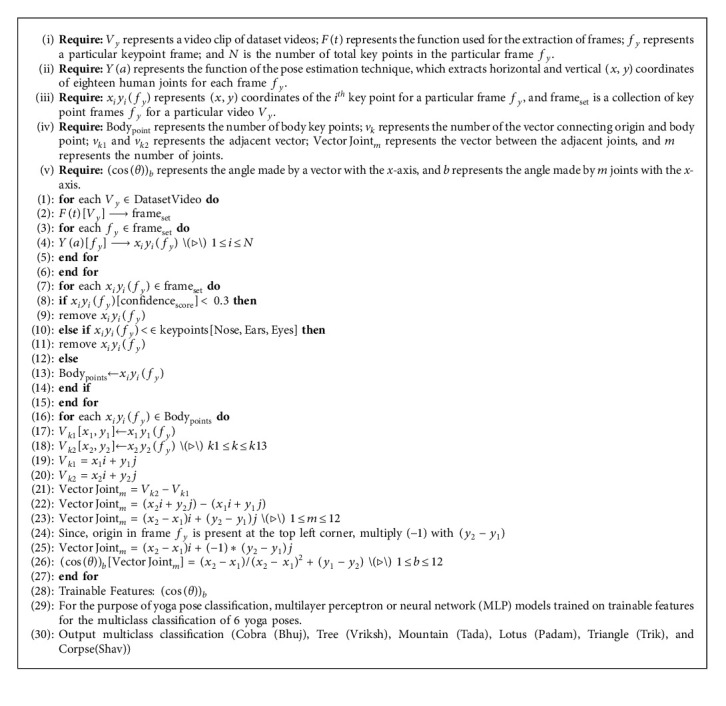
Yoga Pose Classification

**Table 1:** Summarization of statistics of the dataset for each yoga pose.

Yoga pose	Time (s)	Persons	Videos
Cobra pose	615	15	14
Lotus pose	495	15	10
Corpse pose	450	15	10
Mountain pose	585	15	12
Triangle pose	540	15	13
Tree pose	500	15	11
Total			70

**Table 2:** Table represents the accuracy result of the experimented models.

Model	Accuracy	
	Training	Testing
SVM	0.9532	0.9319
CNN	0.9934	0.9858
CNN + LSTM	0.9987	0.9938
MLP	0.9962	0.9958

**Table 3:** Evaluation of proposed work runtime in milliseconds with various techniques running. Mean (F.E.+C) demonstrates the mean average runtime per frame for extraction and computation of features with yoga pose classification. Mean (F.G.) is the mean average runtime per frame for feedback generation.

Methods	CPU		GTX-1080	
	Mean (F.E.+C)	Mean (F.G.)	Mean (F.E.+C)	Mean (F.G.)
SVM	6574.8 ± 134.7	16.4	214.5 ± 10.6	10.32
CNN	6528.6 ± 112.6	15.2	208.4 ± 15.2	8.36
CNN + LSTM	6512.6 ± 118.4	16.6	206.8 ± 16.8	7.18
MLP	6504.4 ± 124.3	12.3	206.3 ± 12.6	6.47

## Data Availability

The data that support the findings are available on request to the corresponding author.

## References

[1] Patil S., Pawar A., Peshave A., Ansari A. N., Navada A. Yoga tutor visualization and analysis using SURF algorithm.

[2] Wu W., Yin W., Guo F. Learning and self-instruction expert system for Yoga.

[3] Chen H. T., He Y. Z., Chou C. L., Lee S. Y., Lin B. S. P., Yu J. Y. Computer-assisted self-training system for sports exercise using kinects.

[4] Trejo E. W., Yuan P. Recognition of Yoga poses through an interactive system with Kinect device.

[5] Mohanty A., Ahmed A., Goswami T., Das A., Vaishnavi P., Sahay R. R. Robust pose recognition using deep learning.

[6] Toshev A., Szegedy C. Deeppose: human pose estimation via deep neural networks.

[7] Cao Z., Simon T., Wei S. E., Sheikh Y. Realtime multi-person 2d pose estimation using part affinity fields.

[8] Cao Z., Hidalgo G., Simon T., Wei S. E., Sheikh Y. (2019). OpenPose: realtime multi-person 2D pose estimation using Part Affinity Fields. *IEEE Transactions on Pattern Analysis and Machine Intelligence*.

[9] Kendall A., Grimes M., Cipolla R. Posenet: a convolutional network for real-time 6-dof camera relocalization.

[10] Kreiss S., Bertoni L., Alahi A. Pifpaf: composite fields for human pose estimation.

[11] Gong W., Zhang X., Gonzàlez J. (2016). Human pose estimation from monocular images: a comprehensive survey. *Sensors*.

[12] Pothanaicker K. (2019). Human action recognition using CNN and LSTM-RNN with attention model. *Intl Journal of Innovative Tech. and Exploring Eng*.

[13] Güler R. A., Neverova N., Kokkinos I. Densepose: dense human pose estimation in the wild.

[14] Chen H.-T., He Y.-Z., Hsu C.-C. (2018). Computer-assisted yoga training system. *Multimedia Tools and Applications*.

[15] Jain S., Rustagi A., Saurav S., Saini R., Singh S. (2021). Three-dimensional CNN-inspired deep learning architecture for Yoga pose recognition in the real-world environment. *Neural Computing & Applications*.

[16] Hsieh C. C., Wu B. S., Lee C. C. (2011). A distance computer vision assisted yoga learning system. *Journal of Computers*.

[17] Uddin M. T., Uddiny M. A. Human activity recognition from wearable sensors using extremely randomized trees.

[18] Jalal A., Sarif N., Kim J. T., Kim T.-S. (2013). Human activity recognition via recognized body parts of human depth silhouettes for residents monitoring services at smart home. *Indoor and Built Environment*.

[19] Zhan Y., Kuroda T. (2014). Wearable sensor-based human activity recognition from environmental background sounds. *Journal of Ambient Intelligence and Humanized Computing*.

[20] Pai P.-F., ChangLiao L.-H., Lin K.-P. (2017). Analyzing basketball games by a support vector machines with decision tree model. *Neural Computing & Applications*.

[21] Haque S., Rabby A. S. A., Laboni M. A., Neehal N., Hossain S. A. ExNET: deep neural network for exercise pose detection.

[22] Palanimeera J., Ponmozhi K. (2021). Classification of yoga pose using machine learning techniques. *Materials Today Proceedings*.

[23] Casas L., Navab N., Demirci S. (2019). Patient 3D body pose estimation from pressure imaging. *International Journal of Computer Assisted Radiology and Surgery*.

[24] Shavit Y., Ferens R. (2019). Introduction to camera pose estimation with deep learning.. https://arxiv.org/abs/1907.05272.

[25] Thang N. D., Kim T.-S., Lee Y.-K., Lee S. (2011). Estimation of 3-D human body posture via co-registration of 3-D human model and sequential stereo information. *Applied Intelligence*.

[26] Uddin M. Z., Thang N. D., Kim T. S. Human activity recognition via 3-D joint angle features and hidden Markov models.

[27] Ofli F., Chaudhry R., Kurillo G., Vidal R., Bajcsy R. (2014). Sequence of the most informative joints (smij): a new representation for human skeletal action recognition. *Journal of Visual Communication and Image Representation*.

[28] İnce Ö.F, Ince I. F., Yıldırım M. E., Park J. S., Song J. K., Yoon B. W. (2020). Human activity recognition with analysis of angles between skeletal joints using a RGB-depth sensor. *ETRI Journal*.

[29] Guler A., Kardaris N., Chandra S. Human joint angle estimation and gesture recognition for assistive robotic vision.

[30] Yadav S. K., Singh A., Gupta A., Raheja J. L. (2019). Real-time Yoga recognition using deep learning. *Neural Computing & Applications*.

[31] Szczuko P. (2019). Deep neural networks for human pose estimation from a very low resolution depth image. *Multimedia Tools and Applications*.

